# Risk assessment of extra-uterine involvement and prognosis in young type I endometrial carcinoma with high or moderate differentiation and less than 1/2 myometrial invasion

**DOI:** 10.18632/aging.205714

**Published:** 2024-04-03

**Authors:** Yi Sun, Pin Han, Yuanpei Wang, Xiaoran Cheng, Weijia Wu, Qianwen Liu, Fang Ren

**Affiliations:** 1 Deparment of Gynecology, The First Affiliated Hospital of Zhengzhou University, Zhengzhou 450000, China; 2 Deparment of Gynecology, The Luoyang Maternal and Child Health Care Hospital, Luoyang 471000, China

**Keywords:** endometrial carcinoma, extra-uterine involvement, lymphadenectomy, differentiation, myometrial invasion

## Abstract

Purpose: The aim of this study was to investigate whether young patients with endometrial carcinoma can preserve adnexa and lymph nodes to improve their quality of life without compromising their prognosis.

Methods: A total of 319 patients with type I endometrial carcinoma (high or moderate differentiation and less than 1/2 myometrial invasion) hospitalized in the First Affiliated Hospital of Zhengzhou University from May 2012 to July 2021 were included. The patients were divided into four groups: high differentiation without myometrial invasion group (G1MI-), high differentiation with superficial myometrial invasion group (G1MI+), moderate differentiation without myometrial invasion group (G2MI-), and moderate differentiation with superficial myometrial invasion group (G2MI+). Logistic regression analysis was conducted to identify risk factors for extra-uterine involvement. Kaplan-Meier method was used to draw the survival curve to compare the prognosis in subgroups and rates of extra-uterine involvement were also compared using Chi-square test or Fisher’s exact test.

Results: Multivariable logistic regression revealed that differentiation (HR = 14.590, 95%CI = 1.778-119.754, *p* = 0.013) and myometrial invasion (HR = 10.732, 95%CI = 0.912-92.780, *p* = 0.037) were the independent risk factors for extra-uterine involvement. The overall difference was statistically significant (*p* < 0.001). In the subgroups analysis, both adnexal metastasis and lymph node metastasis were statistically significant in the G2MI+ group compared with G1MI- (*p* = 0.007, *p* = 0.008). There were no significant differences in the overall survival (OS) rate and progression free survival (PFS) rate among the four subgroups (*p* > 0.05).

Conclusions: Surgery with adnexal preservation and without systematic lymphadenectomy could be employed for the patients who are high differentiation with less than 1/2 myometrial invasion or moderate differentiation without myometrial invasion, but not recommended to the patients with moderate differentiation and superficial myometrial invasion.

## INTRODUCTION

Endometrial carcinoma (EC) is the fourth most common gynecologic malignancy in developed countries for women worldwide, which results in approximately 320,000 new cases each year [1–3]. But with the improvement of living standards and changes in lifestyle, approximately 31% of patients with EC worldwide were overweight or obese which leads to a consequent decrease in exercise and an increase in the incidence of diabetes and hypertension [4, 5]. The above phenomena ultimately lead to an increase in the incidence rate of EC in recent years [6, 7]. The incidence of EC in patients under 40 years of age is around 3-14% [8, 9]. Nowadays, according to National Comprehensive Cancer Network (NCCN) guidelines, the recommend treatment for EC is comprehensive staged surgery (total hysterectomy + bilateral salpingo-oophorectomy ± regional lymphadenectomy) [10]. However, in young female patients, removal of adnexa and lymphadenectomy can lead to a significant impact on quality of life, with significant side effects as well as increased long-term risks, including neurovascular injury, pelvic infection, lymphocysts, cardiovascular disease, osteoporosis and so on [11–13]. Therefore, these young female patients require personalized treatment strategies to improve their quality of life.

Currently, patients diagnosed with EC who can be treated conservatively need to meet the criteria of being high differentiation and without myometrial invasion [8, 14]. The site of metastasis and regional lymph node metastasis (LNM) status of EC patients are important indicators for surgical pathologic staging by the International Federation of Gynecology and Obstetrics (FIGO), as well as important references for evaluating prognosis and guiding treatment [15]. Moreover, LNM and other extra-uterine metastases are highly correlated with the extent of malignant differentiation and the depth of myometrial invasion [16, 17]. However, clinical studies on extra-uterine metastases in young patients with EC are insufficient. Therefore, the question of whether endocrine function can be preserved and whether lymphadenectomy can be performed in young patients with EC remains a serious challenge.

## RESULTS

### Baseline characteristics of enrolled patients

A total of 319 eligible patients were included in this study. At the time of diagnosis, all patients were premenopausal and had no family history of genetic illness. The G1M-group had a median age of 42 (38, 44) years, the G1M+ group had a median age of 41 (37.75, 44) years, the G2MI-group had a median age of 42 (36.50, 43) years, and the G2MI+ group had a median age of 41 (37, 42) years. The baseline characteristics of the included patients were shown in Table 1. There were no significant differences in diabetes, hypertension, live birth, lesion size and Ca125 between different subgroups (*p* > 0.05).

**Table 1:** Baseline characteristics for all patients.

**Characteristics**	**G1MI- n (%)**	**G1MI+ n (%)**	**G2MI- n (%)**	**G2MI+ n (%)**	***p***
**Total number**	152	62	29	72	/
**Median age (years) (P25, P75)**	42 (38,44)	41 (37.75,44)	42 (36.50,43)	41 (37,42)	0.231
**Hypertension/Diabetes**					0.606
Yes	27 (17.76)	8 (12.90)	7 (24.14)	14 (18.42)	
No	125 (82.24)	54 (87.1)	22 (75.86)	62 (81.58)	
**Live birth**					0.075
Yes	121 (79.61)	39 (62.9)	23 (79.31)	56 (73.68)	
No	31 (20.39)	23 (37.1)	6 (20.69)	20 (26.32)	
**Size of lesions**					0.36
<2cm	117 (76.97)	42 (67.74)	24 (82.76)	55 (72.37)	
≥ 2cm	35 (23.03)	20 (32.26)	5 (17.24)	21 (27.63)	
**CA125^a^**					0.206
<35 U/ml	119 (87.50)	43 (81.13)	20 (76.92)	53 (76.81)	
≥35 U/ml	17 (12.50)	10 (18.87)	6 (23.08)	16 (23.19)	

^a^284 people were tested for CA125.

### Risk factors for extra-uterine involvement

A total of 319 eligible patients were included in the study and we divided them into two groups based on extra-uterine involvement. We identified the independent risk factors that may affect extra-uterine involvement using the logistic regression model for univariable and multivariable analysis. As shown in Table 2, differentiation (HR = 14.590, 95%CI = 1.778-119.754, *p* = 0.013) and myometrial invasion (HR = 10.732, 95%CI = 0.912-92.780, *p* = 0.037) were the independent risk factors for extra-uterine involvement.

**Table 2:** Univariable and multivariable logistic regression analysis for extra-uterine involvement.

**Characteristics**	**Univariable analysis**		**Multivariable analysis**	
	**HR (95% CI)**	***p***	**HR (95% CI)**	***p***
**Age**	1.144 (0.959-1.365)	0.136		
**Differentiation**				
Highly	Reference	0.002	Reference	0.013
Moderately	27.484 (3.522-214.452)		14.590 (1.778-119.754)	
**Myometrial invasion**				
No	Reference	0.007	Reference	0.037
Superficial	17.413 (2.201-133.519)		10.732 (0.912-92.780)	
**Tumor Size**				
<2 cm	Reference	0.276		
≥2 cm	1.891 (0.601-5.956)			
**Ca125**				
<35 U/ml	Reference	0.935		
≥35 U/ml	0.975 (0.538-1.769)			

### Differences in physiology of type I young EC patients

### Overall difference of extra-uterine involvement in four groups

Since no patients had vaginal, parastatal, peripheral or distant metastases, the rates of cervical stroma metastases, serosal/adnexal metastases, pelvic lymph node metastases, para-aortic LNM and external of corpus uteri invasion in the subgroups were compared, respectively (Table 3). The results showed that there was no statistical difference in the rate of cervical stroma involvement and para-aortic LNM among the four subgroups, with *p*-values of 0.524 and 0.587, respectively. The rates of serosal/adnexal metastasis, pelvic LNM and total metastasis of the four subgroups were statistically significant difference (*p* = 0.006, *p* = 0.003, *p* < 0.001).

**Table 3:** Pathological characteristics of subgroups.

**Metastatic sites**	**G1MI-n (%)**	**G1MI+n (%)**	**G2MI-n (%)**	**G2MI+n (%)**	***p***
**Total metastasis**					
Yes	0	1 (1.61)	1 (3.45)	11 (14/47)	<0.001
No	152 (100)	61 (98.39)	28 (96.55)	65 (85.53)	
**Cervical stromal**					
Yes	0	0	0	1 (1.32)	0.524
No	152 (100)	62 (100)	29 (100)	75 (98.68)	
**Serous layer/Adnexa**					
Yes	0	1 (1.61)	1 (3.45)	5 (6.58)	0.006
No	152 (100)	61 (98.39)	28 (96.55)	71 (93.43)	
**Pelvic lymph node^a^**					
Yes	0	0	0	5 (8.06)	0.003
No	117 (100)	53 (100)	20 (100)	57 (91.94)	
**Abdominal aortic lymph node^b^**					
Yes	0	0	0	1 (2.27)	0.587
No	64 (100)	35 (100)	12 (100)	43 (97.73)	

^a^252 cases underwent total hysterectomy + bilateral salpingo-oophorectomy + regional lymphadenectomy.

^b^155 cases underwent total hysterectomy + bilateral salpingo-oophorectomy + pelvic lymphadenectomy + para-aortic lymphadenectomy.

The patients in G1MI- group without extra-uterine metastases. With the rate (1.61%, 1/62) of extra-uterine involvement in the G1MI+ group, postoperative pathology of one patient showed that the uterine cavity was full of cauliflower-like neoplasm and cancer tissue was visible on the left ovarian surface, which was considered as metastasis.

The rate of extra-uterine invasion of G2MI- group was 3.45% (1/29). The patient underwent TH + BSO + PL + PAL under laparoscopy, and pathology indicated focal endometrial cancer, with moderate differentiation endometrioid adenocarcinoma visible on the surface of the right ovary, attributed to metastasis.

The rate of extra-uterine invasion of G2MI+ group was 14.47% (11/76). There were 5 cases involving the serous layer of the uterus or adnexa. Among the 62 patients who underwent uterine and bilateral salpingo-oophorectomy plus regional lymphadenectomy, there were 5 patients had pelvic lymph node metastasis, and 1 patient was accompanied by para-aortic lymph node metastasis. In the G2MI+ group, there was no patients with distant metastasis.

### Pairwise comparison of serosal/adnexa metastasis rate

The serosal/adnexa metastasis rate of the patients in the three subgroups (G1MI+, G2MI-, and G2MI+) was compared with the patients in G1MI- group who were able to preserve the adexa and lymph nodes (Table 4).

**Table 4:** Pairwise comparison of different characteristics' metastasis rate.

**Group\Characteristics**	**Serosal/adnexa metastasis rate**					**Pelvic LNM rate**					**Extra-uterine involvement rate**			
	**Fourfold table (n)**		**χ2**	***p***		**Fourfold table (n)**		**χ2**	***p***		**Fourfold table (n)**		**χ2**	***p***
**G1MI- vs G1MI+**	152	0	-	0.29		117	0	-	-		152	0	-	0.29
	61	1				53	0				61	1		
**G1MI- vs G2MI-**	152	0	-	0.16		117	0	-	-		152	0	-	0.16
	28	1				20	0				28	1		
**G1MI- vs G2MI+**	152	0	7.387	0.007		117	0	6.964	0.008		152	0	15.977	<0.001
	71	5				57	5				65	11		

The Bonferroni method was used to correct the test level α as 0.0167. The result showed that there was no significant difference compared with G1MI+ group and G2MI- group (*p* = 0.290, *p* = 0.160). However, the difference between the G2MI+ group and the G1MI - group was statistically significant (*p* = 0.007).

### Pairwise comparison of pelvic lymph node metastasis rate

As there were no patients with positive pelvic lymph nodes in the G1MI+ and G2MI- groups, only pairwise comparison was made on the rate of pelvic LNM in the G1MI- and G2MI+ groups, and the Bonferroni correction test level α was 0.0167. The results showed that the difference was statistically significant (*p* = 0.008) (Table 4).

### Pairwise comparison of external of total metastasis

The rates of extra-uterine involvement for G1MI+, G2MI- and G2MI+ groups were compared with G1MI- group individually, and the Bonferroni correction test level α was 0.0167. The results showed that G1MI+ and G2MI- groups were not significantly different from that of G1MI- group (*p* = 0.290, *p* = 0.160). The difference between G2MI+ group and G1MI- group was significant (*p* < 0.001), as shown in Table 4.

### Survival outcomes of patients

**Figure 1:**
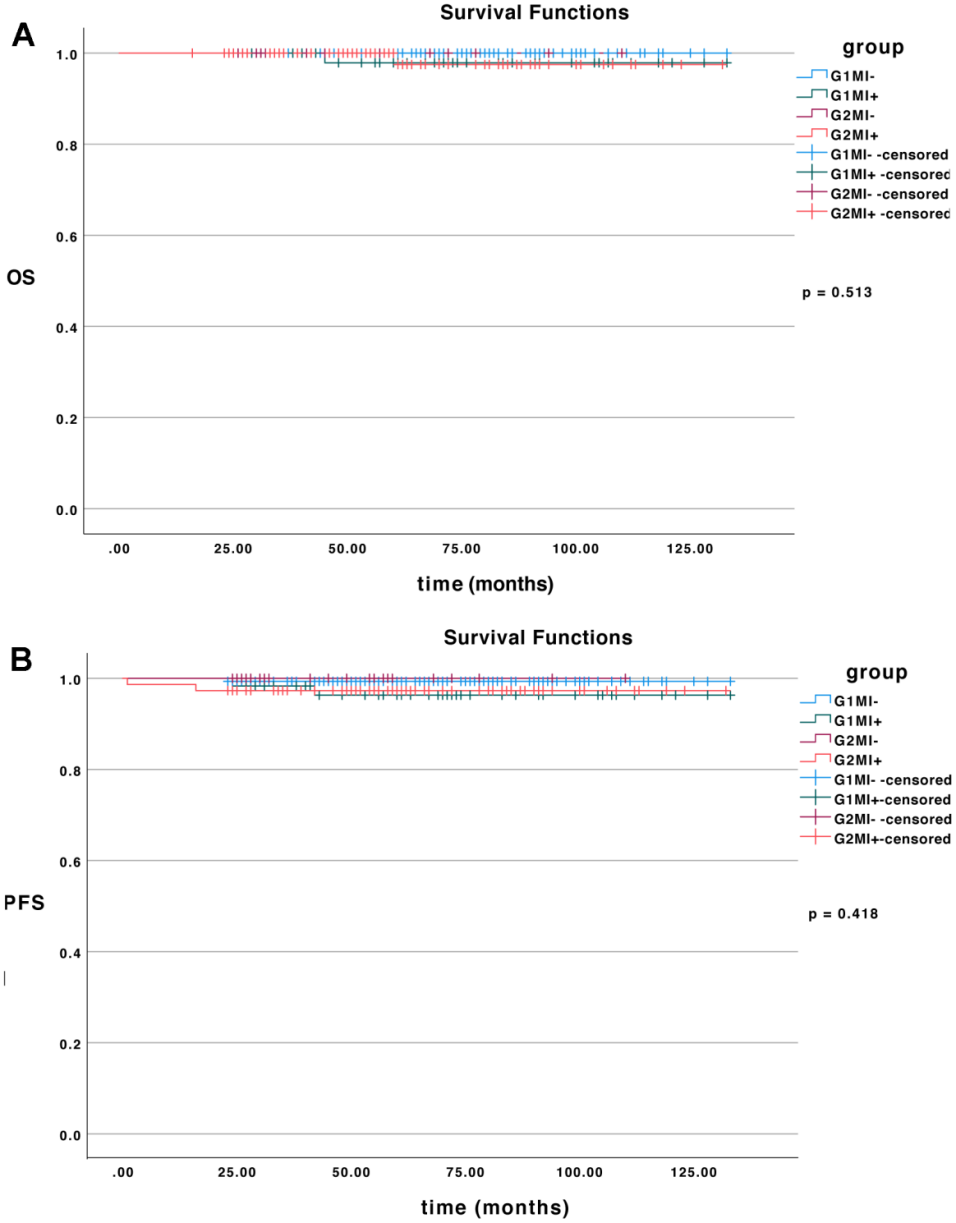
**The survival curves.** (**A**) OS of all patients in different subgroups (*p* = 0.513). (**B**) PFS of all patients in different subgroups (*p* = 0.418).

The Kaplan-Meier method was used to calculate the OS and PFS. There were 149, 59, 27, and 74 patients with prognosis in the four subgroups, respectively. There were 10 patients lost to follow-up. The results showed that the PFS rates did not differ among the four subgroups (*p* = 0.418), which were 99.3%, 96.6%, 100% and 97.3% respectively (Figure 1A). There were also no significant differences in the four subgroups regarding OS rates (*p* = 0.513), which were 100%, 98.3%, 100% and 98.6% respectively (Figure 1B).

## DISCUSSION

In our study, we measured the effects of various characteristics on extra-uterine involvement in young EC patients with high or moderate differentiation and less than 1/2 myometrial invasion. Differentiation and myometrial invasion were identified as two independent risk factors for extra-uterine involvement through multivariable logistic regression analysis. Among these patients, young EC patients with G1MI- are suitable population for conservative treatment without surgery. In our study, patients in the G1MI- group had no extra-uterine involvement (0/152), while the rates of extra-uterine metastasis in the G1MI+ group, G2MI- group, and G2MI+ group were 1.61% (1/62), 3.45% (1/29), and 14.47% (11/74), respectively. And the difference was statistically significant (*p* < 0.001).

The 2021 European Society of Gynaecological Oncology (ESGO) criteria [11] for the preservation of adnexa include the following: age < 45 years, low-grade endometrioid carcinoma, myometrial invasion depth < 1/2, absence of ovarian and other extra-uterine lesions. Studies have suggested that differentiation and myometrial invasion are independent risk factors for adnexal involvement [18–20]. The results of our article are consistent. The rates of adnexal metastasis in the four groups were 0, 1.61%, 3.45%, and 6.58%, respectively, and the difference was statistically significant (*p* = 0.006). We determined that only the difference between the G2MI+ group and the G1MI-group was statistically significant (*p* = 0.007) by doing a further subgroups analysis of the results. There was no statistically significant difference between G1MI- and G2MI-. In addition, some studies have suggested that preserving the ovaries have no impact on the prognosis of premenopausal EC patients [21, 22]. The analysis of the survival prognosis in this study revealed that even though plasma membrane and adnexal metastases occurred, there was no statistically significant difference in the PFS and OS of the patients. Studies have shown that approximately 20% of EC patients are premenopausal at the time of diagnosis, and 5% are under the age of 40 [8, 9], which means that more EC patients are focusing on prolonged survival as well as quality of life [23]. Therefore, the above findings provide a theoretical basis for patients in the G1MI-, G1MI+, and G2M- groups to retain their adnexa.

Most young patients with EC present with early-stage disease, and almost 80% have tumors confined to the uterus [24]. The degree of tumor differentiation and the depth of myometrial invasion are established risk factors for regional LNM [16, 17, 25, 26]. The GOG 33 study showed that the pelvic LNM rates were 3%, 9%, and 18% for patients with high, moderate, and poor differentiation, respectively (*p* < 0.0001), and the para-aortic LNM rates were 2%, 5% and 11% (*p* < 0.0007), respectively [16]. In the GOG 33 study, the metastatic rates of pelvic and para-aortic lymph nodes without myometrial invasion were both 1%, with superficial invasion at 5% and 3%, and deep invasion at 25% and 17%. Moreover, Mariani et al. conducted a retrospective analysis and found that patients with high or moderate differentiation, myometrial invasion <1/2 and the tumor size of < 2 cm had a lower risk of LNM [27].

In this study, it was found that there was no statistical difference in LNM among subgroups, only the G2MI+ group developed LNM (5/62). The difference between G2MI+ group and G1MI- group was statistically significant (*p* = 0.008). A large study comparing 250 patients found that systematic lymphadenectomy only improved surgical staging and did not improve patients' OS and PFS [28]. Our current efforts aim to improve the quality of life of young female patients without compromising prognosis. Moreover, lymphadenectomy may result in neurovascular injury, pelvic infection, lymphocyst and lymphedema formation [29]. Therefore, patients in the G1MI-, G1MI+, and G2M- groups should be carefully considered for systematic lymphadenectomy after fully assessing their disease.

Molecular classification is correlated with prognosis, clinical management, and the personalization of patient therapy [30, 31]. The use of molecular classification has provided a significant advantage by enabling the precise selection of patients who benefit from systemic treatments [32, 33]. Moreover, molecular analysis could also be used to determine the therapeutic strategy for the conservative treatment of lesions that anticipate EC [34, 35]. Zhang et al. conducted a retrospective analysis of 59 patients with EC and endometrial atypical hyperplasia/endometrial intraepithelial neoplasia (EAH/EIN) to assess how molecular classification could predict response to conservative treatment and identify the subclasses at the highest risk of evolution [34]. Therefore, if it is possible to stratify the management of patients by molecular classification, the treatment of patients with EC will be more precise. However, molecular classification was not included in our data, so we look forward to subsequent related studies.

This study possesses notable strengths. Based on our findings, it is possible to provides a theoretical basis for individualized treatment of young patients with EC, which would help to improve the efficiency of treatment and mitigate unnecessary risks. However, it also possesses limitations. Firstly, this study was a single-center retrospective study with some bias in the data, and more prospective studies are needed to confirm the accuracy of the findings. Secondly, the current technical tools for determining differentiation and myometrial invasion still have limitations, and enhancing accuracy remains a clinical challenge. Moreover, the missing of molecular aspects in these patients was another limitation that should be addressed in the future.

This study found that the patients with high differentiation and less than 1/2 myometrial invasion or moderate differentiation without myometrial invasion could preserve adnexa and avoid systemic lymphadenectomy under the premise of sufficient risk notification and clinical follow-up. In cases of moderate differentiation with superficial myometrial invasion, considering the survival prognosis was not significantly decreased, the rate of extra-uterine involvement was significantly increased, preserving adnexa and not performing systematic lymphadenectomy are still not recommended yet. And more prospective data are needed for further support in the future.

## MATERIALS AND METHODS

### Patient selection

Prior to its initiation, this study was reviewed and approved by the Ethics Committee of the First Affiliated Hospital of Zhengzhou University. We retrospectively reviewed our laboratory information system database for women who underwent surgery due to EC from May 2012 to July 2021. We ultimately included 319 patients with EC who met the following criteria.

The inclusion criteria are as follows: (1) 18 ≤ age ≤ 45 years; (2) the treatment methods included total hysterectomy (TH) + bilateral salpingo-oophorectomy (BSO) ± pelvic lymphadenectomy (PL) ± para-aortic lymphadenectomy (PAL); (3) the postoperative pathological evidence was confirmed by two or more senior pathologists: endometrial carcinoma, high or moderate differentiation and the lesion was confined to the endometrium or involved only the superficial muscular layer, that is, <1/2 myometrial invasion.

The exclusion criteria are as follows: (1) adjuvant therapy before operation; (2) patients with a history of other malignant tumors; (3) physiology was confirmed as double primary carcinoma of endometrium and ovary by two or more senior pathologists; (4) missing or incomplete data.

### Grouping and evaluation indicators

According to the postoperative pathological report, the enrolled patients were divided into 4 groups: 152 patients in the high differentiation without myometrial invasion group (G1MI-), 62 patients in the high differentiation with superficial myometrial invasion group (G1MI+), 29 patients in the moderate differentiation without myometrial invasion group (G2MI-) and 76 patients in the moderate differentiation with superficial myometrial invasion group (G2MI+). Among these four groups, G1MI- is the indication population for fertility preservation treatment for those who are able to preserve the adnexa and lymph nodes.

Comparing the rates of extra-uterine invasion and survival prognosis among subgroups, extra-uterine invasion includes involvement of cervical stroma, serous layer, adnexa, vagina, parametrium, regional lymph nodes, bladder or rectum invasion, and distant metastases. The primary study metrics for survival analysis were progression free survival (PFS) and overall survival (OS). PFS was the time from the date of treatment to tumor recurrence or the last follow-up time; OS was the time from the day of treatment to death or last follow-up. The deadline of follow-up was August 1, 2023.

### Statistical analysis

Statistical analysis was performed using GraphPad Prism 8.0 and SPSS 26.0. The quantitative data submitted to the normality test were expressed as mean ± standard deviation for normal distribution and median for non-normal distribution. The Pearson Chi-square test, continuous correction Chi-square test or Fisher exact test were used to compare the qualitative data of independent groups. α = 0.05 was used as the test level, and *p* < 0.05 was considered statistically significant. Continuous correction Chi-square test or Fisher's exact test was used for pairwise comparison. The test level α was corrected by Bonferroni method, with κ representing the number of pairwise comparisons, taking 3 as the value. The significance level was set at *p* < α/κ, resulting in *p* < 0.0167, indicating a statistically significant difference. Logistic regression analysis of risk factors for extra-uterine involvement was performed. Kaplan-Meier method was used to draw the survival curve, and the difference of survival curve was compared by Log-rank test.
